# Novel mutation in the SETD1A gene in a newborn patient associating with congenital airway and heart defeats: A case report

**DOI:** 10.1097/MD.0000000000033449

**Published:** 2023-03-31

**Authors:** Long Jin, Wendi Mo, Yu Yan, Ying Wang

**Affiliations:** a Department of Respiratory Medicine, Anhui Provincial Children’s Hospital, Hefei, Anhui, China.

**Keywords:** airway, case report, congenital, mutation, SETD1A

## Abstract

Congenital malformations are commonly associated with genetic disorders in infants and children.

**Patient concerns:**

A 13-day-old male infant was admitted to our hospital presenting with aggravated dyspnea and characteristic facial and body features. Examinations during hospitalization found congenital bronchomalacia and heart defects including atrial septal defect, patent ductus arteriosus, and pulmonary hypertension, congenital laryngeal stridor, and tracheal stenosis.

**Diagnosis:**

Considering complicated clinical manifestations, the Trio Whole Exon Sequencing test was run to screen for any hereditary diseases and found a heterozygous pathogenic mutation in SET domain containing 1A (SETD1A) gene (c.2096T > A; p. Leu699Ter, 1099), which was a *de novo* mutation.

**Intervention:**

The patient was given amoxicillin clavulanate potassium for antibiotic, fibro bronchoscope lavage and other symptomatic support therapy, and referred to the department of Cardiac Surgery for arterial catheter ligation.

**Outcomes:**

The patient was discharged after postoperative recovery without shunt. In the following 2 years, he was admitted to hospital multiple times during to infectious pneumonia.

**Lessons:**

SETD1A gene mutation is commonly associated with neuropsychiatric disorders. This is the first reported case with a novel mutation of SETD1A gene along with new associated phenotypes. Our results broaden the genotypic and phenotypes spectrum of SETD1A gene mutation in infant patients.

## 1. Introduction

Genetic disorders have commonly been the major contributor to the development of congenital diseases in neonates and children. For example, it was reported that around 1 third of congenital heart disease are attributed to genetic disorders, concurrent with the presentations of malformations of other organs.^[1]^ The well-known and common congenital diseases proved to be caused by a single gene disorder in China including glucose-6-phosphate dehydrogenase deficiency, congenital hypothyroidism, phenylketonuria, hemophilia and thalassemia.^[2]^ For rare genetic disorders, it is often more challenging for clinicians to recognize and treat them properly. Uncovering gene mutations via genetic testing and establishing its associated phenotype spectrum is crucial for understanding the pathogenesis of multiple congenital diseases and facilitate the relevant treatment strategies.

SET domain containing 1A (SETD1A) is a gene that has been shown in previous studies to be needed from the early stage in development for epigenetic control of the gene expression and maintaining genome stability.^[3]^ Pathogenic variants in SETD1A, encoding SET Domain Containing 1A, was previously reported that was involved in the development of a broad range of neuropsychiatric disorders including schizophrenia, developmental delay/intellectual disability and autism spectrum disorders.^[4,5]^ Takata et al^[6]^ identified 2 *de novo* loss-of-function variants in the SETD1A gene by analyzing exome sequencing data from 231 patients with schizophrenia and 34 control trios. However, no studies have so far reported the congenital malformations that affect airway and cardiovascular system that was correlated to SETD1A mutation. We therefore here present the case of a male infant who were found to have a novel mutation site in SETD1A, which was considered to attribute to its congenital malformations that affects respiratory and cardiovascular systems.

## 2. Case presentation

A male infant on his 13th day after delivery was referred to the Department of Respiratory Medicine, Anhui Provincial Children’s Hospital, for aggravated dyspnea with crying over the last 7 days. The baby was delivered by natural labor at 39 weeks, with a birth weight of 3400 grams and Apgar score of 10 to 10. There were no perinatal asphyxia and rescue after delivery. The patient was the third child of healthy non-consanguineous Chinese parents. His siblings are healthy and had no major health issues including developmental delay and other neuropsychic disorders. His father was 35 years old and his mother was 31 years old at his birth.

After admission, on examination, the patient was found to have polypnea (90 times/minute), stridor, and characteristic facial and body features including upward slant of the eyes, ocular hypertelorism, webbed neck, and broad, short hands with a single crease in the palm (also known as single transverse palmar crease). The cardiac auscultation found muffled cardiac sounds and grade 3 systolic murmurs. Heart rate was 152 times per minutes. Reflections can be elicited.

**Figure 1.**
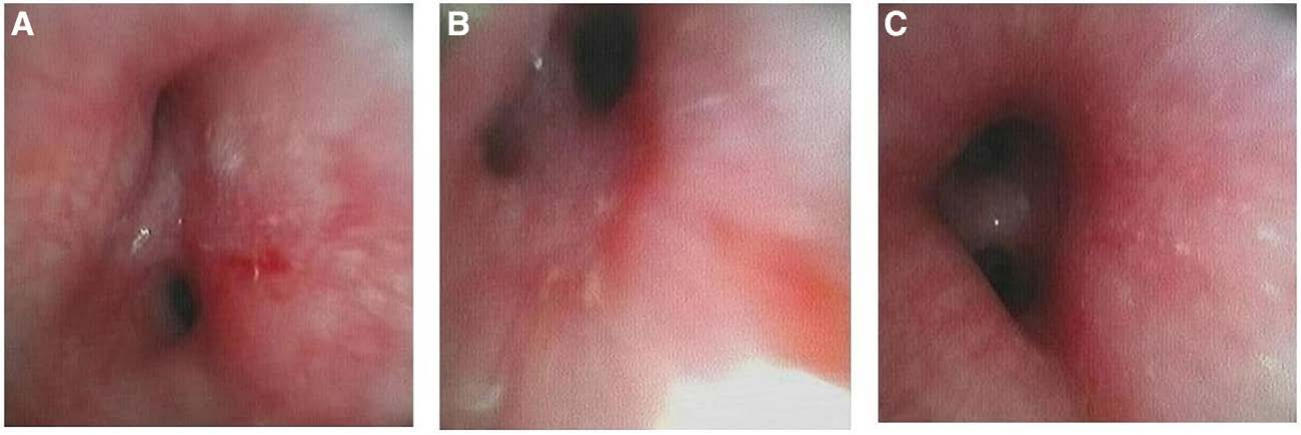
Bronchoscopic findings. (A) Shows that the left upper bronchus was moderately narrowed, with marked mucosal hyperemia and 50% depression of the wall seen on inhalation, (B) shows moderate narrowing of the left lower bronchus with mucosal congestion and viscous discharge, and (C) shows mild malacia of the distal left main bronchus, with depression up to 25% on inhalation.

Echocardiography was performed while the infant was resting. It revealed that the patient had left ventricular (LV) enlargement (LV diastolic dimension: 2.2cm; LV systolic dimension: 1.3cm), widened pulmonary artery, atrial septal defect (secondary foramen) of 0.29 cm, patent ductus arteriosus about 0.36 cm, accompanied by pulmonary hypertension; There was no enlargement of aortic artery. The fraction shortening value was 38% and ejection fraction was 71%, which were in the normal range. The valve activity and blood flow velocity were normal. Chest X-ray showed that the lungs are blurred with a few patchy and dense shadows. Head magnetic resonance imaging did not find abnormality; Chromosome karyotype was 46 X.Y. The Fragile X syndrome screening test found no abnormalities. After admission, the fiberoptic bronchoscope was given, which showed a retroverted epiglottis, incomplete glottis closure, malacia of the distal left main bronchus, and moderate stenosis of the left upper and lower lobe broncho trachea (Fig. 1). Laboratory investigations found increased lymphocyte count, decreased red blood cell count, decreased hemoglobin, and increased platelet count. No remarkable abnormities were found in liver and renal functions, and thyroid functions, nor was there abnormity in screening test for hereditary hematuria.

The initial diagnoses were as follows: neonatal pneumonia, congenital bronchomalacia, congenital heart disease (atrial septal defect, patent ductus arteriosus, and pulmonary hypertension), congenital laryngeal stridor, and tracheal stenosis.

Given that the clinical manifestations are congenital and heterogeneous, we decided to run genetic tests to screen for any hereditary diseases. We obtained the informed consent from the proband and his parents for undertaking the Trio Whole Exome Sequencing. Deoxyribonucleic acid was extracted from the peripheral blood. The coding exons were captured through the xGen Exome Research Panel v2.0 (IDT) (www.idtdna.com). The suspected pathological mutations were further verified using Sanger sequencing.

The Whole exome sequencing test found a heterozygous truncating mutation in exon 8 of the SETD1A gene of the proband, at base 2096. The T was mutated to A, changing thymine to adenine (c.2096T>A, p.L699*,1909), leading to the mutation of the amino acid residue at position 699 from leucine to a stop codon. As the parents did not carry the same genetic mutation nor they had similar symptoms as the patient, this mutation was a *de novo* mutation. This mutation was rare and had a relatively low distribution frequency in the population. The mutation was considered as a pathogenic mutation (PVS1 + PS2 + PM2) according to the 2015 variant classifications of the American College of Medical Genetics and Genomics Standards and Guidelines.^[7]^ This mutation was not found in dbSNP (http://www.ncbi.nlm.nih.gov/projects/SNP/) and Genome (http://www.internationalgenome.org/) databases. The genetic pattern of the mutation fits autosomal dominant inheritance. Results of the Trio Whole Exome Sequencing are shown in Table 1. According to the patient’s clinical manifestations and testing results of genomics, a diagnosis of *de novo* SETD1A mutation was made.

**Table 1:** Findings of the whole exome sequencing test.

Gene	Chromosomal location	Nucleic acid change (exon no.)	Amino acid changes	RS No.	ACMG pathogenicity grade	Proband (male)	Father (other phenotype)	Mother (normal)	Related diseases (OMIM), genetic patterns
SETD1A	CHR 16:30977298	c.2096 (exon8) T > A	p. L699*,1009 (p. Leu699Ter,1009)(NM_014712)	None	Pathogenic	Heterozygosity(64/121)	Wild type (0/79)	Wild type (0/66)	Early onset epilepsy with or without growth retardation (OMIM: 618832), AD
KCNK4	CHR11:64067057	c.1041 (exon7)C > T	p. I347I (p. I1e347=)(NM_033310)	RS773117171	Possibly pathogenic	Heterozygosity	Wild type	Wild type	Facial deformities, hypertrichosis, epilepsy, mental/developmental retardation and gingival hypergrowth syndrome (OMIM: 618381), AD
CDK13	CHR7:40132675	c.3527 (exon13)A > G	p. Q1176R (p. G1n1176Arg)(NM_003718)	None	Uncertain	Heterozygosity	Wild type	Heterozygosity	Congenital heart defects, facial deformities and mental development disorders (OMIM:617360), AD
CHD7	CHR8: 61732567	c.2615 (exon9)T > C	p. I872T (p. I1e872Thr)(NM_017780)	RS751181139	Possibly pathogenic	Heterozygosity	Wild type	Heterozygosity	Charge Syndrome (OMIM: 214800), AD
HPGD	CHR4: 175413160	c.748 (exon7)G > C	p. G250R (p. Gly250Arg)(NM_000860)	None	Uncertain	Heterozygosity	Wild type	Heterozygosity	Congenitally isolated clubbing finger (OMIM: 119900), AD, AR
MUC5B	CHR11:1249993	c.974 (exon8)G > A	p. C325Y (p. Cys325Tyr)(NM_002458)	None	Uncertain	Heterozygosity	Heterozygosity	Wild type	Idiopathic pulmonary fibrosis, susceptible (OMIM: 178500), AD
HRAS	CHR11:532704	c.502 (exon5)C > G	p. L168V (p. Leu168Val)(NM_001130442)	RS766474932	Uncertain	Heterozygosity	Wild type	Heterozygosity	Facio-cutaneous-skeletal syndrome (OMIM: 218040), AD
TMEM67	CHR8:94808191	c.1836 (exon18)T > G	p. Y612*,384 (p. Tyr612Ter,384)(NM_153704)	None	Pathologic	Heterozygosity	Heterozygosity	Wild type	Renal wasting disease type 11 (OMIM: 613550), AR;Meckel syndrome type 3 (OMIM:607361), AR;RHYNS syndrome; RHYNS (OMIM: 602152), AR;COACH syndrome (OMIM: 216360), AR
TNXB	CHR6: 32049924	c.3625 (exon9)G > T	p. E1209*, 2945 (p. Glu1209Ter, 2945)(NM_019105)	None	Possibly pathogenic	Heterozygosity	Heterozygosity	Wild type	Vesicoureteral reflux (OMIM: 615963), AD

ACMG = American College of Medical Genetics and Genomics, AD = autosomal dominant, AR = autosomal recessive, OMIM = online mendelian inheritance in man, SETD1A = SET domain containing 1A.

The patient was given amoxicillin clavulanate potassium (30 mg q 8 hours) for antibiotic and other symptomatic support therapy for 5 days and were well-tolerated, fibro bronchoscope lavage was given after 3 days antibiotic treatment, and then referred to the department of Cardiac Surgery for arterial catheter ligation after the symptoms were well-controlled. He was then discharged after postoperative recovery without shunt. On his 2 years old follow-up, he weighed 14 kg and the height is 85 cm, both within the normal range. He has been hospitalized for recurrent infectious pneumonia since her birth, and the most common pathogens include Streptococcus pneumoniae, Haemophilus influenzae and mycoplasma. There were no obvious developmental, psychological or behavioral disorders during the growth of the patient, and no convulsions of any form has been found so far. The electroencephalogram and head magnetic resonance imaging were normal. Epilepsy and other neurological and psychological manifestations were not observed and recorded.

## 3. Discussion and conclusion

Congenital heart defects and malformations of airway are the 2 leading causes of mortality in infants and children and understanding the underlying pathology and etiology is important for making timely and appropriate treatment decisions and preventing the progression of diseases. Over the last 2 decades with the advancement of genetic technologies, the development of congenital diseases has been frequently linked and attributed to genetic disorders. In this study, we have reported a case of infant who were found to have both abnormal morphology of heart and respiratory systems, which were associated with a novel truncation mutation in SETD1A gene. During the treatment, there was no adverse event or anticipants events occurring.

Mutations in SETD1A gene was reported that are commonly associated with epilepsy, developmental delay, and other neuropsychological and behavioral disorder in pediatric patients (OMIM: 618832). The role of the SETD1A gene mutation in affecting the morphology of respiratory and cardiovascular systems has not yet been found and reported. In this paper, we reported an infant case who was found to have a novel mutation in the SETD1A gene that has never been recorded before. Phenotypes including bronchial isomerism and cardiac abnormality associated with this mutation are also novel and distinct from the phenotypes that are seen in previous literature. The case reported in our study did not show growth retardation, developmental delay, epilepsy, schizophrenia or other psychological and behavioral abnormalities till 2 years old. However, it cannot be ruled out that the patient is too young to develop these diseases and present with the corresponding symptoms. The new phenotypes with the novel mutation in SETD1A gene reported in this case indicates that both genotypic and phenotypic spectrum with SETD1A mutation can be much wider than what has been reported in previous literature. More studies and case reports are needed to confirm the casual relationship between SETD1A mutation and congenital anomalies in cardiovascular and respiratory systems, and explore the underlying biological mechanisms of highly penetrant genetic mutations like SETD1A mutation as reported in our report leading into multiple developmental disorders. This will be important to improve our understanding of the disease pathogenesis and assist in making appropriate treatment strategies.

In conclusion, this is the first reported case with a novel mutation of SETD1A gene presenting with new associated phenotypes. This case improves our understanding of the developmental abnormalities associated with SETD1A mutations and broaden the genotypic and phenotypes spectrum of SETD1A gene mutation, thus provides valuable information for clinical diagnosis and genetic counseling to this genetic disorder.

## Author contributions

**Conceptualization:** Long Jin, Wendi Mo, Yu Yan, Ying Wang.

**Writing – original draft:** Long Jin, Ying Wang.

**Writing – review & editing:** Wendi Mo, Yu Yan.
